# Long non-coding RNAs and mRNAs profiling during spleen development in pig

**DOI:** 10.1371/journal.pone.0193552

**Published:** 2018-03-14

**Authors:** Tiandong Che, Diyan Li, Long Jin, Yuhua Fu, Yingkai Liu, Pengliang Liu, Yixin Wang, Qianzi Tang, Jideng Ma, Xun Wang, Anan Jiang, Xuewei Li, Mingzhou Li

**Affiliations:** 1 Institute of Animal Genetics and Breeding, College of Animal Science and Technology, Sichuan Agricultural University, Chengdu, Sichuan, China; 2 School of Computer Science and Technology, Wuhan University of Technology, Wuhan, Hubei, China; Kunming University of Science and Technology, CHINA

## Abstract

Genome-wide transcriptomic studies in humans and mice have become extensive and mature. However, a comprehensive and systematic understanding of protein-coding genes and long non-coding RNAs (lncRNAs) expressed during pig spleen development has not been achieved. LncRNAs are known to participate in regulatory networks for an array of biological processes. Here, we constructed 18 RNA libraries from developing fetal pig spleen (55 days before birth), postnatal pig spleens (0, 30, 180 days and 2 years after birth), and the samples from the 2-year-old Wild Boar. A total of 15,040 lncRNA transcripts were identified among these samples. We found that the temporal expression pattern of lncRNAs was more restricted than observed for protein-coding genes. Time-series analysis showed two large modules for protein-coding genes and lncRNAs. The up-regulated module was enriched for genes related to immune and inflammatory function, while the down-regulated module was enriched for cell proliferation processes such as cell division and DNA replication. Co-expression networks indicated the functional relatedness between protein-coding genes and lncRNAs, which were enriched for similar functions over the series of time points examined. We identified numerous differentially expressed protein-coding genes and lncRNAs in all five developmental stages. Notably, ceruloplasmin precursor (*CP*), a protein-coding gene participating in antioxidant and iron transport processes, was differentially expressed in all stages. This study provides the first catalog of the developing pig spleen, and contributes to a fuller understanding of the molecular mechanisms underpinning mammalian spleen development.

## Introduction

The developmental complexity of organisms arises from elaborate gene regulation rather than an increase in the number of protein-coding genes [1]. Systematic transcriptome analyses by deep sequencing of human cell lines have revealed that ~75% of the human genome is transcribed into primary transcripts, but less than 3% of the genome accounts for protein-coding transcripts [2]. This means that the vast majority of the mammalian genome generates non-coding RNAs (ncRNAs). Long non-coding RNAs (lncRNAs) are defined as ncRNAs with a length longer than 200 nt. It has been proposed that lncRNAs may serve as versatile regulators of diverse aspects of biology in physiological and pathological contexts [3,4]. At present, a large number of lncRNAs has been discovered in mammals [5–9], and many play an important role in biological processes such as X-chromosome inactivation [10], genomic imprinting [11], disease [3], cell differentiation and development [3,12].

Pigs (*Sus scrofa*) are domesticated in multiple centers across Eurasia [13,14], where they are important for agricultural economics. To date, over ten genomes of pig breeds with distinct phenotypes have been published [15–19], constituting a huge resource to understand whole genome characteristics of this species. Moreover, pigs have been employed as a biomedical model for research on human diseases [20–22]. Spleen, the secondary lymphoid organ, has functions in iron metabolism, immunization and hematopoiesis [23]. However, very little transcriptomic research on pig spleen has been published. To improve the robustness and resilience of pigs against pathogens through selection, a better understanding of transcriptomic factors involved in the immune response is required [24].

Here, we report the systematic identification and characterization of lncRNAs in pig spleen during five developmental stages using an Illumina HiSeq platform. A total of 15,040 lncRNAs were identified. To the best of our knowledge, no other report describing spleen lncRNAs and their biological function in pigs is currently available. Our results not only provide a useful resource for better understanding the regulatory functions of lncRNAs in pig and annotation of the pig genome, but also they contribute to better comprehension of mammalian spleen development.

## Materials and methods

### Animals and sample collection

Three healthy female pigs (Rongchang pig, a Chinese indigenous breed in Rongchang, Chongqing) were examined at each of the five developmental stages in this study, including E55d (55 days before birth), B0d (just after birth), W30d (weaned for 30 days), A180d (180 days of age) and A2Y (2 years of age). In addition, three 2-year old female Wild Boars (WB) were included, which were collected from Ya’an, Sichuan. The spleen was rapidly dissected from each carcass and immediately frozen in liquid nitrogen. All samples were stored at −80°C until total RNA extraction. Animals were humanely killed to ameliorate suffering by intravenous injection with 2% pentobarbital sodium (25 mg/Kg). All experimental procedures and sample collection in this study were approved by the Institutional Animal Care and Use Committee (IACUC) of Sichuan Agricultural University, under permit No. DKY-B20141401.

### RNA-seq

Total RNA was isolated using a standard Trizol (Invitrogen) protocol. Genomic DNA was removed using DNaseI. Eighteen strand-specific cDNA libraries for paired-end 150 bp sequencing were prepared using dUTP protocols. Libraries were sequenced on Illumina’s HiSeq platform. More than 10 Gb of high quality data were obtained per library.

### Identification of lncRNAs

To obtain high quality lncRNAs, we replaced the heterosome of the reference genome with the newest version [25]. The filtered clean reads were mapped to the new reference genome using Tophat version 2.0.11 [26]. Transcripts were assembled by Stringtie version 1.3.3 [27]. Transcripts with lengths < 200 nt were filtered. Next, assembled transcripts from each sample were merged into a consensus transcriptome using previously published custom Python scripts [8]. Cuffcompare version 2.2.1 [28] was used to remove the transcripts that were annotated in the reference as “c” and “=“ (“c” for partial match, and “=“ for full match). Remaining transcripts that contained a known protein-coding domain were removed by Hmmscan [29] and BLASTX. The Coding Potential Calculator (CPC) [30] was used to assess the coding potential of the remaining transcripts, and transcripts with CPC score > 0 were removed. Finally, the remaining transcripts with FPKM > 0 at least in one biological replicate were annotated as lncRNAs.

### Classification of lncRNAs

LncRNAs were classified based on their genomic characterization by FEELnc [31]. The resulting set of lncRNAs was subdivided into five categories: (1) no overlap with other loci, classified as intergenic lncRNAs (lincRNAs); (2) overlap with sense intron; (3) overlap with antisense intron; (4) overlap with sense exon; and (5) overlap with antisense exon. The last four classes include two conditions: (a) the lncRNA contains the intron or exon; (b) the lncRNA is contained within the intron or exon.

### Expression analysis

Stringtie version 1.3.3 were applied to quantify protein-coding genes and lncRNAs expression, and obtained FPKM expression values (denoted as fragments per kb of transcript per Mb of mapped reads). FPKM > 0.1 was used to filter the expressed protein-coding genes [32]. Log_2_ transformed values of (FPKM+1) were used in subsequent analyses. Pearson correlations were calculated across developmental stages. Principal Component Analysis (PCA) was carried out using R.

The Shannon entropy (*H*) [33] is calculated as:

$$H_{g}\;=\;\sum_{1\leq t\leq N}{-P}_{t/g}{\;\text{log}}_{2}\left(P_{t/g}\right)$$

where *P*_*t/g*_ is the relative expression of a gene *g* in a stage *t* relative to its expression given in *N* stages. This value has units of bits ranging from zero, indicating genes expressed in a single stage, to log_2_(*N*), indicating genes expressed uniformly in all developmental stages considered. DESeq2 [34] was applied to detect differentially expressed protein-coding genes and lncRNAs based on the read count produced from FPKM by Stringtie version 1.3.3. Benjamini-adjusted *P* values ≤ 0.05 were identified as DEGs.

### Time-series analysis

Time-series analysis was performed by STEM (Short Time-series Expression Miner) [35]. Significantly enriched model profiles are indicated by different colors (Bonferroni-adjusted *P* values ≤ 0.05). Model profiles of the same color belong to the same cluster of profiles.

### Co-expression networks

A co-expression network was constructed across the five developmental stages by R package WGCNA [36] to analyze sets of protein-coding genes and lncRNAs that were significant for each time point, as evaluated by the R package DESeq2 with a time-series model.

### PSI estimation

Values for PSI (percent spliced in) were calculated as previously described [37]. The PSI value of each annotated exon was obtained at every developmental stage. PCA and Pearson correlations were performed to analyze expression.

### Functional enrichment analyses for genes

The DAVID (Database for Annotation, Visualization and Integrated Discovery) web server was used to perform functional enrichment analysis of Gene Ontology (GO) and KEGG pathway categories. Genes were mapped to their respective human orthologous and the resulting list was submitted to DAVID for enrichment analysis of significant overrepresentation of GO biological processes (GO-BP), molecular function (GO-MF) terminologies, and KEGG pathway categories. During the analysis, the whole gene set was treated as the background. Only terms with Benjamini-adjusted *P* values ≤ 0.05 were considered significant.

## Results

### Identification of lncRNAs in pig spleen

To systematically identify lncRNAs and their spatiotemporal expression profiles during spleen development in pig, we constructed 18 cDNA libraries for strand-specific, paired-end 150 bp sequencing on Illumina’s HiSeq platform, representing five important developmental stages: E55d (55 days before birth), B0d (just after birth), W30d (weaned for 30 days), A180d (180 days old) and A2Y (2 years old, including Wild Boar). On average, we obtained about ~37.03 Gb of high quality data per stage (S1 Table). We developed a highly stringent step-wise protocol to discard transcripts not possessing all the characteristics of lncRNAs (S1 Fig). We identified putative lncRNAs by considering their homology with known proteins, containment of a known protein-coding domain, and coding potential. In total, we obtained a “high-confidence” set of 15,040 lncRNAs in pig spleen, each of which was expressed in at least one biological replicate (FPKM > 0) (S2 Table). We found that 13,047 lncRNAs existed in WB. Among them, 195 lncRNAs were specific to WB, which were not identified in other domestic pig samples in our study.

### Genomic characterization and classification of lncRNAs

Compared with protein-coding genes, lncRNAs are shorter in length, have fewer exons, and are expressed at lower levels (S2A–S2C and S3 Figs). These phenomenons all exist in domestic pigs and WBs, which are consistent with previous studies in mammals [38–40]. According to their genomic location, results of FEELnc analysis for the best lncRNA-mRNA partner interaction of each of 12,188 identified lncRNAs could be partitioned into five groups: 9,063 intergenic lncRNAs (lincRNAs) without any gene overlap, 1,195 sense exon-overlapping lncRNAs, 568 antisense exon-overlapping lncRNAs, 268 sense intron-overlapping lncRNAs, and 1,094 antisense intron-overlapping lncRNAs (S2D Fig). Functional enrichment analysis was performed on genes whose exon or intron overlapped with lncRNAs. The results showed that T cell activation involved in immune response (GO:0002286), humoral immune response (GO:0006959), adaptive immune response (GO:0002250), and blood coagulation (GO:0007596) were significantly enriched for genes with intron-overlapping lncRNAs (S3 Table).

### Expression profiles of protein-coding genes and lncRNAs

**Fig 1:**
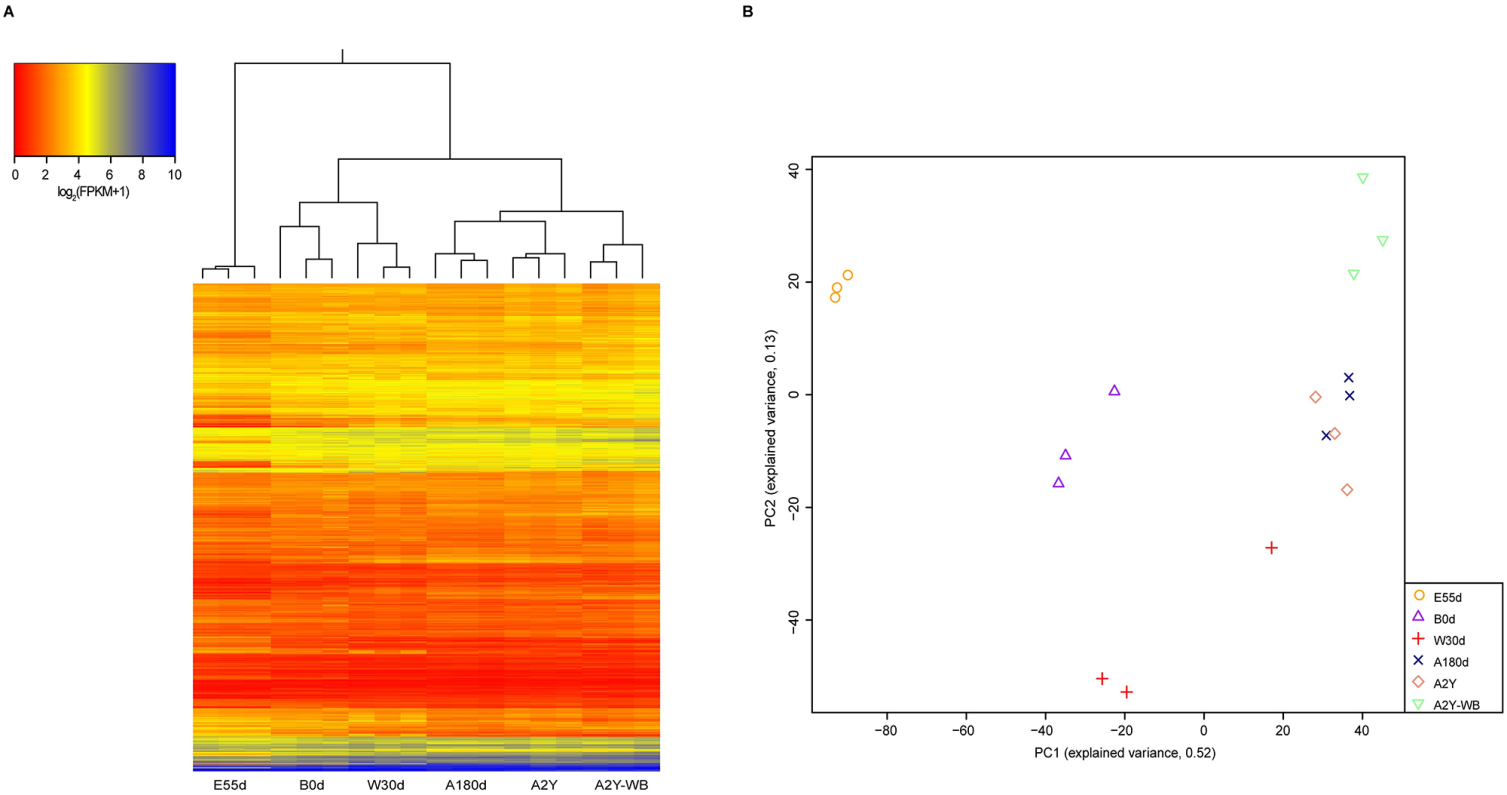
Expression profile and PCA of protein-coding genes. (A) Heat map showing the expression profile of protein-coding genes. The top panel is the tree constructed by Pearson correlation. (B) Two-way PCA plot of protein-coding genes based on expression profile.

**Fig 2:**
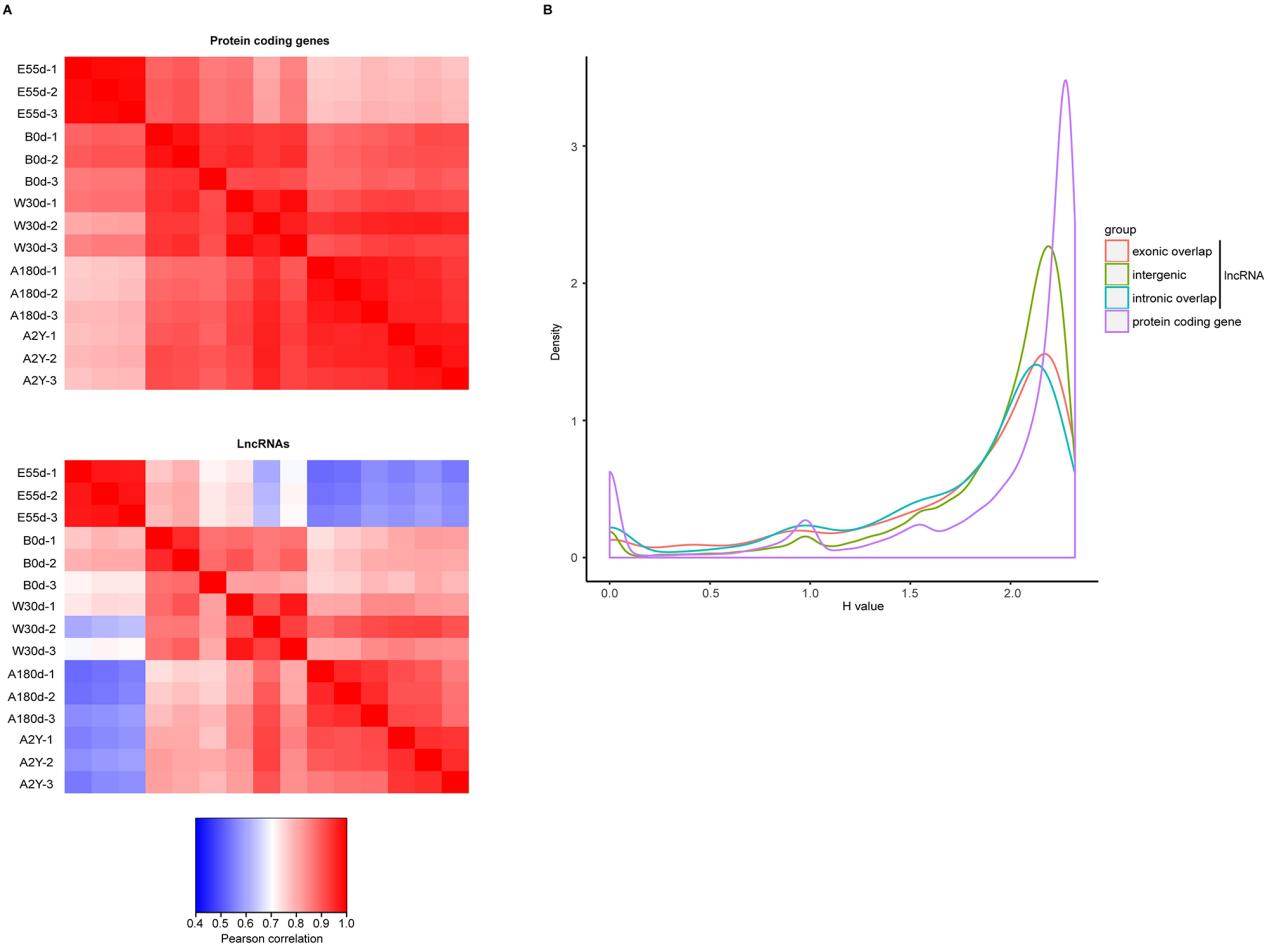
Temporal expression profiles of protein-coding genes and lncRNAs. (A) Dynamic changes in expression profiles of protein-coding genes and lncRNAs. The top panel shows protein-coding genes and the bottom panel shows lncRNAs. Values represent the pairwise Pearson correlation. Correlation between every two samples was calculated by log_2_-transformed (FPKM+1) gene expression values. Three main expression patterns can be distinguished. (B) Distributions of Shannon entropy-based temporal specificity scores were calculated for distinct classes of lncRNAs and protein-coding genes.

Based on expression profiles (S2 and S4 Tables), we observed that samples not only could be separated by different developmental stages, but also could be distinguished between A2Y domestic pig and WB, indicating that both protein-coding genes and lncRNAs are expressed in a stage-specific manner (Fig 1 and S4 Fig). To follow the expression dynamics of lncRNAs and protein-coding genes as development proceeds, we calculated Pearson correlations between each pair of samples based on the expression profiles of all five developmental stages. This resulted in the discovery of two interesting phenomenon. First, comparison of independently clustered expression profiles of samples revealed that both protein-coding genes and lncRNAs could be grouped into three broad classes (Fig 2A): (1) samples from the embryonic development stage (E55d) were congregated, (2) samples from the suckling period (B0d and W30d) were clustered, and (3) samples from the late stage of development (A180d and A2Y) formed the last group.

Second, the power of correlation between two consecutive stages was weaker for lncRNAs than for protein-coding genes. This observation implies that lncRNAs have a more restricted temporal expression than protein-coding genes. To further validate this hypothesis, we calculated the Shannon entropy (*H*) value as a measure of the specificity of gene expression across developmental stages. All three classes of lncRNAs (lincRNAs, and intron-overlapping and exon-overlapping lncRNAs) showed increased temporal specificity compared with protein-coding genes (Fig 2B).

### Time-series analysis and co-expression network of protein-coding genes and lncRNAs

**Fig 3:**
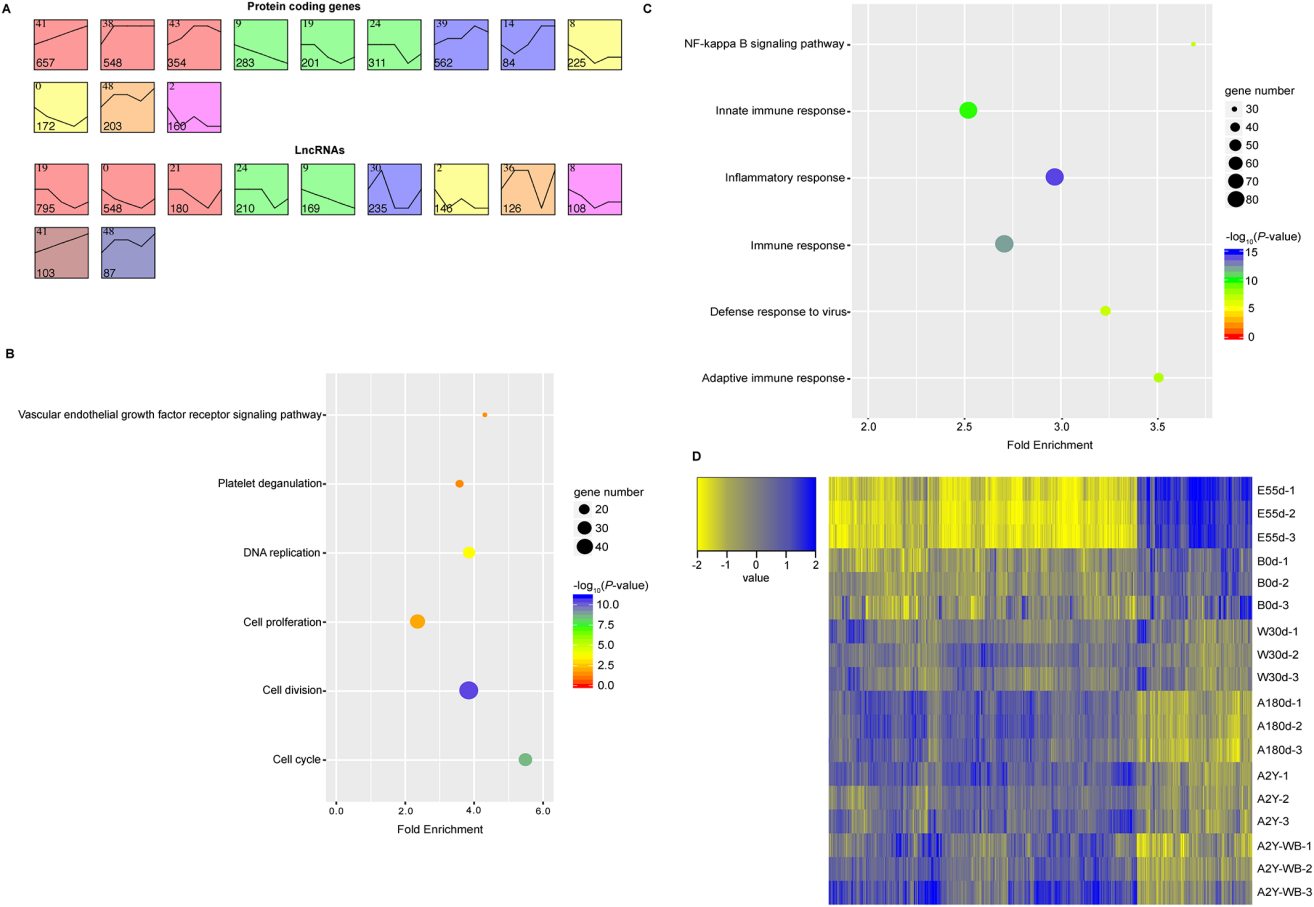
Time-series modules and co-expression network of lncRNAs and protein-coding genes. (A) Time-series modules of protein-coding genes and lncRNAs. The top panel shows protein-coding genes and the second panel shows lncRNAs. Numbers in the top left corner indicate module number. Numbers in lower left corners indicate numbers of protein-coding genes or lncRNAs in each module. The same color was used to represent each cluster. Functional categories of genes in green (B) and red modules (C). Benjamini adjusted *P* values were transformed by ‒log_10_. (D) Heat map showing the largest two co-expression networks of protein-coding genes. Values represent log_2_(FPKM+1) of each gene in each sample minus the mean value of each gene across all samples.

To explore the dynamic expression pattern of protein-coding genes and lncRNAs, we adopted a time-series analysis by STEM (Short Time-series Expression Miner). According to their dynamic expression patterns across the five development stages, we found that 12,913 protein-coding genes and 15,040 lncRNAs were classified into six and eight cluster profiles, respectively, which included 12 and 11 significantly enriched model profiles (colored in figure, Bonferroni adjusted *P* values ≤ 0.05), respectively (Fig 3A, S5 and S6 Tables). To our surprise, eight model profiles existed in both protein-coding genes and lncRNAs, which indicated that their expression patterns during developmental stages were highly correlated. This implies functional relatedness or a regulatory relationship between protein-coding genes and lncRNAs. Combining gene number and significance level, we used the genes in red (increased expression level with time) and green (decreased expression level with time) modules to perform functional enrichment analysis. Interestingly, genes in green modules were mainly enriched for cell division (GO:0051301) and DNA replication (GO:0006260) (Fig 3B and S7 Table), while genes in red modules were mainly enriched for immune response (GO:0006955) and inflammatory response (GO:0006954) (Fig 3C and S8 Table). This finding is consistent with the process of spleen development.

To explore the functional relatedness between protein-coding genes and lncRNAs, we used co-expression analysis. First, we screened out the set of protein-coding genes and lncRNAs that were related with the factor of time by DESeq2. The differentially expressed protein-coding genes and lncRNAs between A2Y domestic pig and WB were also included. Next, we built the co-expression network based on this set of protein-coding genes and lncRNAs by WGCNA. FPKM > 0.1 was used as the cutoff to filter the protein-coding genes and lncRNAs. A total of 5,474 protein-coding genes and 4,223 lncRNAs were used to construct the co-expression network. Finally, we observed 11 modules and only considered the two largest modules and the overlapping genes (Fig 3D and S9 Table), which accounted for 73.23% of total genes in the 11 modules. Functional enrichment analysis showed that the co-expressed genes in the two largest modules were enriched in a variety of biological processes. Some of them were related to immune response (GO:0006955), inflammatory response (GO:0006954), protein binding (GO:0005515), cell proliferation (GO:0008283) and DNA replication (GO:0006260) (S10 Table).

### Identification of differentially expressed protein-coding genes and lncRNAs

**Fig 4:**
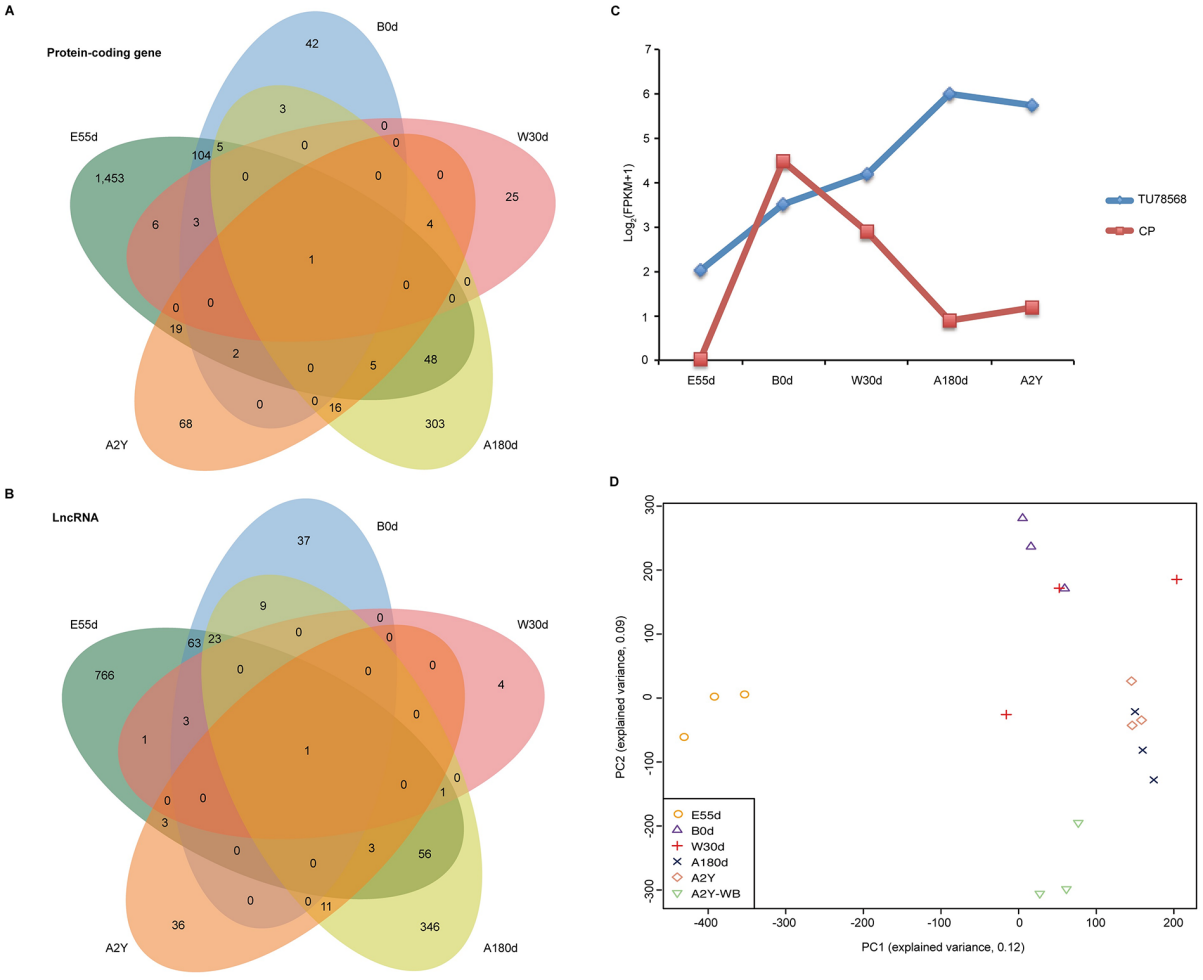
Differentially expressed protein-coding genes and lncRNAs, and PCA of PSI values. Venn diagram of common differentially expressed protein-coding genes (A) and lncRNAs (B) in five developmental stages. (C) Dynamic expression profiles of *CP* and TU78568. (D) Two-way PCA plot of protein-coding genes based on PSI values.

To explore the biological function of each stage, we performed pairwise comparisons between the five developmental stages. First, we identified differentially expressed protein-coding genes and lncRNAs between one stage and each of the other four stages (S11 and S12 Tables). Next, we merged the former protein-coding genes and lncRNAs into a non-redundant set for each stage. A Venn diagram was constructed using the non-redundant set (Fig 4A and 4B). Only one protein-coding gene and lncRNA was differentially expressed in all five developmental stages. This protein-coding gene, *CP*, encodes ceruloplasmin precursor, which has ferroxidase activity to oxidize Fe^2+^ to Fe^3+^ without releasing radical oxygen species, suggesting it may be related to antioxidant processes [41]. Interesting, it is also involved in iron transport across the cell membrane, which is consistent with the main biological function of spleen [23]. In addition, we also found that the expression level of *CP* was almost zero at E55d and reached a maximum at B0d. Afterwards, it gradually declined and remained stable throughout the process of growth and development (Fig 4C). Compared with *CP*, expression of the lncRNA (TU78568) gradually increased across all five stages (Fig 4C), suggesting it may play an important role in spleen development. However, it is disappointing that this lncRNA is located on the scaffold (GL895479.1), where there is no protein-coding gene in the adjacent position.

Functional enrichment analysis was performed on stage-specific differentially expressed protein-coding genes. Genes at E55d were significantly enriched for immune response (GO:0006955) and inflammatory response (GO:0006954) (S13 Table). No significant enrichment for biological functions was observed during the other four stages, which may result from the lack of gene number. Next, non-redundant differentially expressed protein-coding genes for each stage were analyzed for functional enrichment. E55d and B0d were enriched for terms related to immunity and inflammation (S14 Table). Differentially expressed protein-coding genes between WB and A2Y domestic pig were enriched for terms related to protein binding and hematopoietic cell lineages (S15 Table).

### Splicing landscape of protein-coding genes

To explore alternative splicing levels of protein-coding genes across developmental stages, we performed analyses to calculate “percent spliced in” (PSI) values for exons, similar to analysis performed for gene expression levels. Consistent with the results of expression profiling, alternative splicing could also separate samples by different developmental stages, including domestic pig and WB (Fig 4D), indicating that splicing levels have spatiotemporal specificity as well. Although the power of alternative splicing levels to distinguish populations was not stronger than gene expression profiles, individual differences were more distinguishable at the level of splicing.

## Discussion

In this study, we developed a highly stringent filtering pipeline to identify lncRNAs (S1 Fig). In total, we identified 15,040 lncRNAs in the developing pig spleen. To the best of our knowledge, this is the first report to systematically identify lncRNAs during pig spleen development using RNA-seq data.

According to our results, protein-coding genes were highly temporally restricted in terms of both expression profiles and levels of alternative splicing, indicating the function of protein-coding genes is stage-specific [42]. Compared with protein-coding genes, lncRNAs were expressed during narrower time windows (Fig 2B), indicating lncRNAs were more temporally restricted than protein-coding genes [43]. Time-series analysis indicated that both protein-coding genes and lncRNAs are dynamically expressed during spleen development and have similar expression patterns. These results suggest consistent biological function between protein-coding genes and lncRNAs. Genes in red modules with up-regulated expression levels were enriched for genes related to immune and inflammatory responses, such as *TNF*, *IL18*, *IL15*, *IL10* and *IL7R* [44, 45]. Among of these genes, the *TNF* superfamily plays an important role in immunization and inflammation [46, 47]. Genes in green modules with down-regulated expression levels were enriched for biological functions associated with cell division and DNA replication, such as the *CDC* family and *CDK6* [48]. In addition, we also observed two GO terms, vascular endothelial growth factor receptor signaling pathway (GO: 0048010) and platelet degranulation (GO:0002576) (S7 Table), which implied the spleen might participate in the process of hematopoiesis [49] during the process of development. Functional analysis of co-expressed genes showed similar results, further confirming this biological function of the spleen. *CP* was differentially expressed in all five developmental stages, with expression levels ranging from almost none to maximum during the first two stages. B0d represented the moment when the pig birthed and the environment changed from womb to natural atmosphere. This process involves the piglet encountering a high amount of oxygen, which may activate antioxidant mechanisms. *CP* can oxidize Fe^2+^ to Fe^3+^ without releasing radical oxygen species, and is related to antioxidant processes. Its expression level gradually declined and tended to remain stable at subsequent stages, suggesting *CP* might participate in iron transport during these stages. This is also consistent with the splenic function of filtering blood and recycling iron from aging red blood cells [23].

In our study, we added the sample of WB, which enriched our data and made us obtain more lncRNAs in pig spleen. Based on our results, the character of lnRNAs in WB was consistent with domestic pig. Besides, according to the expression profiles of protein-coding genes and lncRNAs, WBs were clustered together with the A180d and A2Y domestic pigs but not separated into a single one, which implied that both protein-coding genes and lncRNAs had stronger stage-specific than breed-specific.

In summary, our study provides the first catalog of lncRNAs in the developing pig spleen. Our results suggest numerous roles of lncRNAs in spleen development and provide a high quality resource for future transcriptomic, genetic and genomic studies.

## Supporting information

S1 Fig Identification pipeline of lncRNAs. (TIF)

S2 Fig Genomic characterization of lncRNAs. (A) Distribution of transcript length for lncRNAs and protein-coding genes. (B) Exon number distribution of lncRNAs and protein-coding genes. (C) Comparison of the expression levels of lncRNAs and protein-coding genes. (D) Classification of lncRNAs.
(TIF)

S3 Fig Comparison of the expression levels of lncRNAs and protein-coding genes. (TIF)

S4 Fig Expression profile and PCA of lncRNAs. **(A)** Heat map shows the expression profile of lncRNAs. The top panel shows the tree constructed by Pearson correlation. **(B)** Two-way PCA plot of lncRNAs based on expression profile.
(TIF)

S1 Table Summary of samples for total RNA sequencing. (DOCX)

S2 Table The identified lncRNAs in pig spleen. (XLSX)

S3 Table Functional categories of genes of intronic overlapping lncRNAs. (XLSX)

S4 Table The log_2_-transformed (FPKM+1) of protein-coding genes. (XLSX)

S5 Table Protein-coding gene list in different model profiles of STEM. (XLSX)

S6 Table LncRNA list in different model profiles of STEM. (XLSX)

S7 Table Functional categories of genes in green modules. (XLSX)

S8 Table Functional categories of genes in red modules. (XLSX)

S9 Table Protein-coding gene and lncRNA list in co-expression network. (XLSX)

S10 Table Functional categories of genes in co-expression network. (XLSX)

S11 Table Differentially expressed protein-coding genes in pairwise stages. (XLSX)

S12 Table Differentially expressed lncRNAs in pairwise stages. (XLSX)

S13 Table Functional categories of genes specific differentially expressed in E55d. (XLSX)

S14 Table Functional categories of genes differentially expressed in E55d and B0d. (XLSX)

S15 Table Functional categories of genes differentially expressed between WB and domestic pig. (XLSX)
