# Supplementary figures and images for: Long non-coding RNAs and mRNAs profiling during spleen development in pig
Source: PLoS One. 2018 Mar 14;13(3):e0193552. doi: 10.1371/journal.pone.0193552 (PMC5851557; doi:10.1371/journal.pone.0193552)

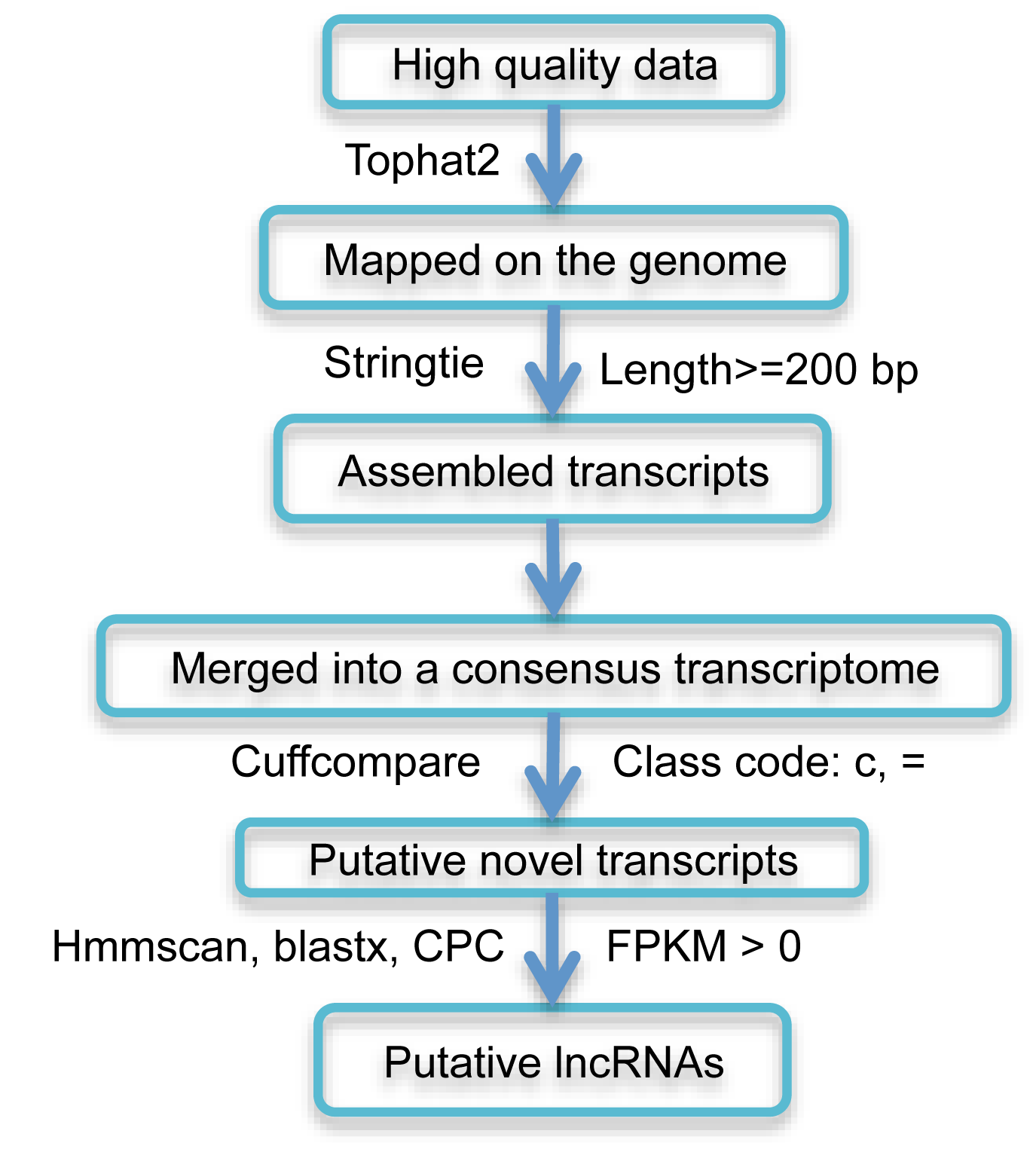

Supplement: S1 Fig — (TIF) [file pone.0193552.s001.tif]

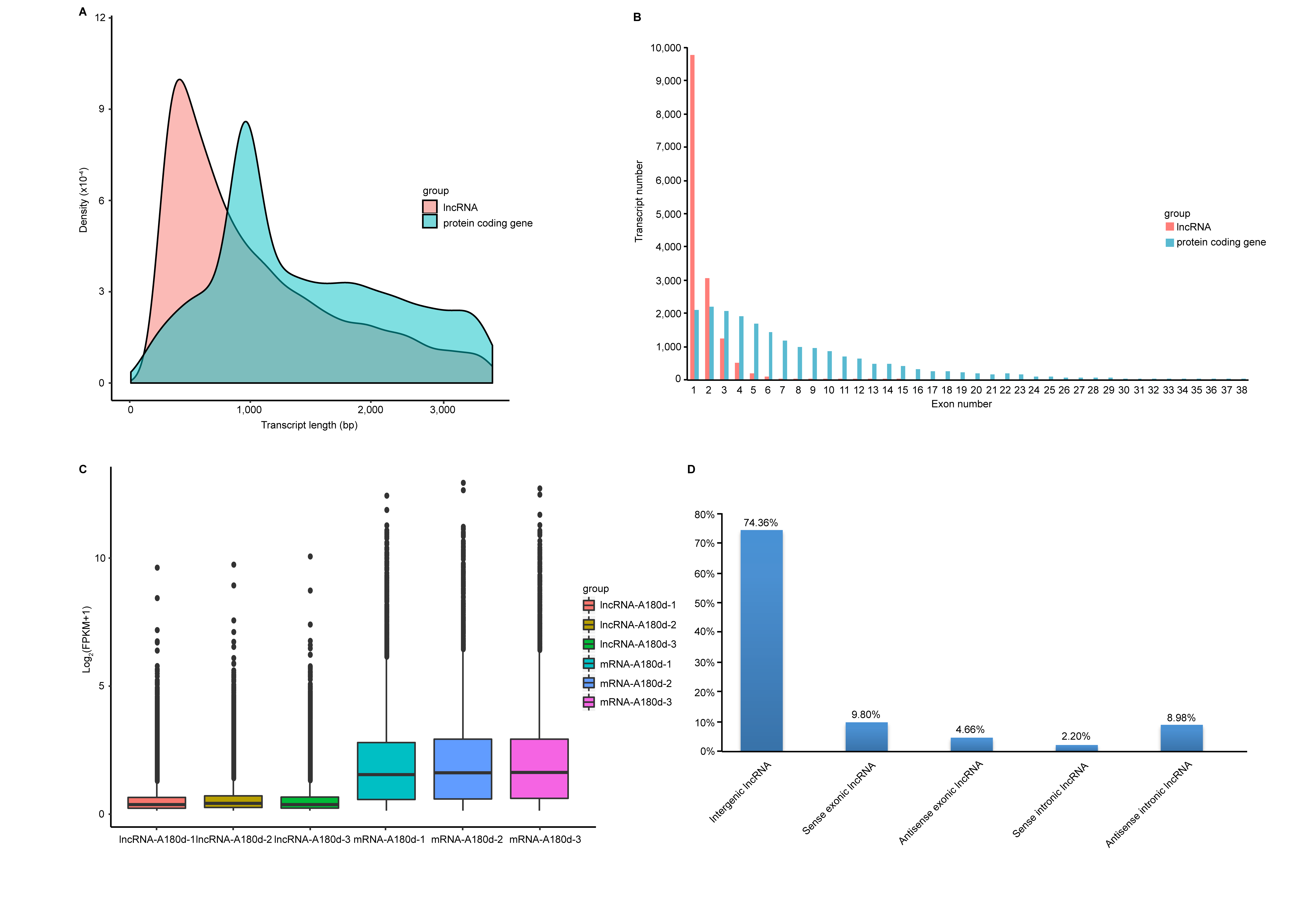

Supplement: S2 Fig — (A) Distribution of transcript length for lncRNAs and protein-coding genes. (B) Exon number distribution of lncRNAs and protein-coding genes. (C) Comparison of the expression levels of lncRNAs and protein-coding genes. (D) Classification of lncRNAs. (TIF) [file pone.0193552.s002.tif]

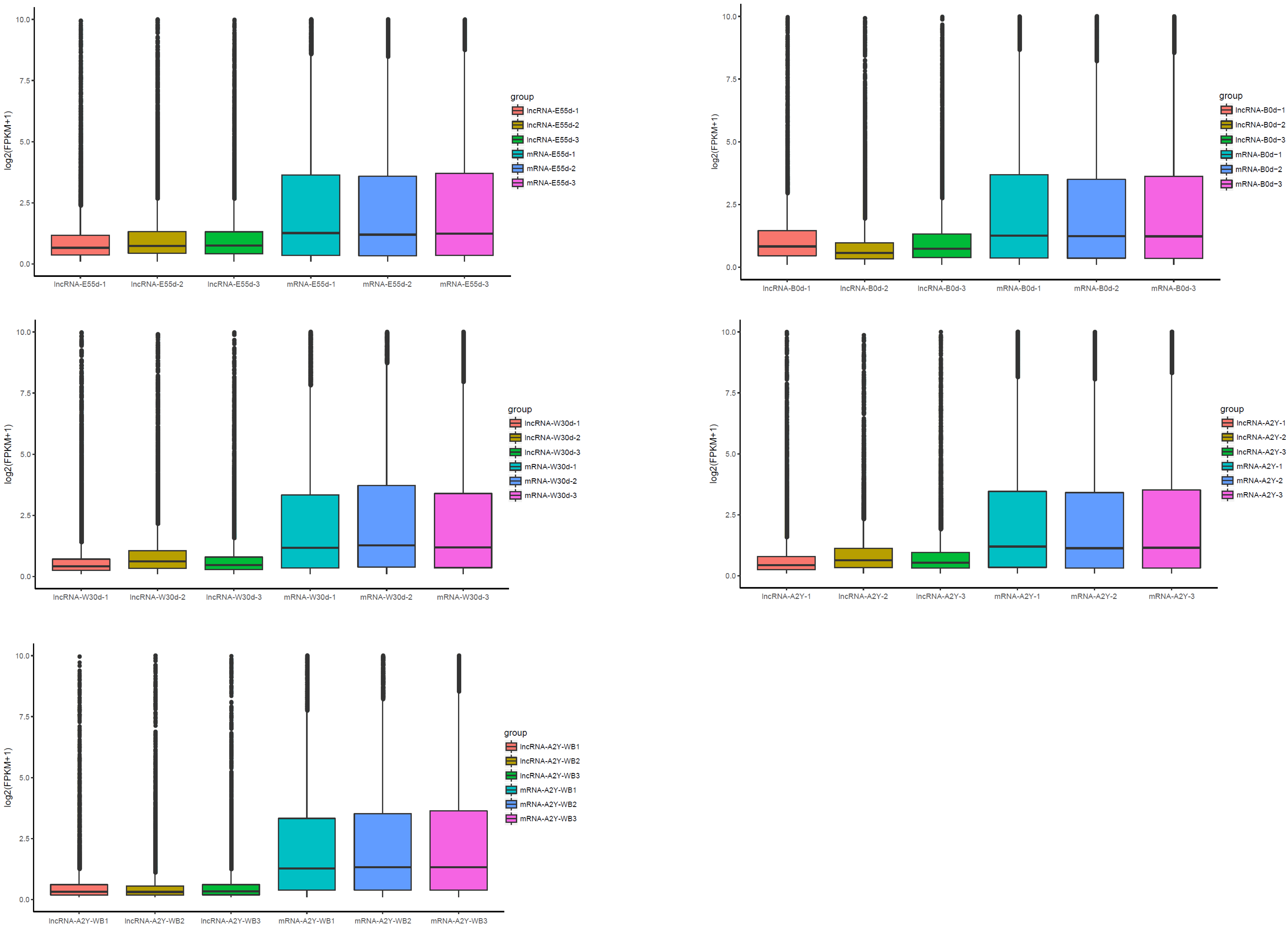

Supplement: S3 Fig — (TIF) [file pone.0193552.s003.tif]

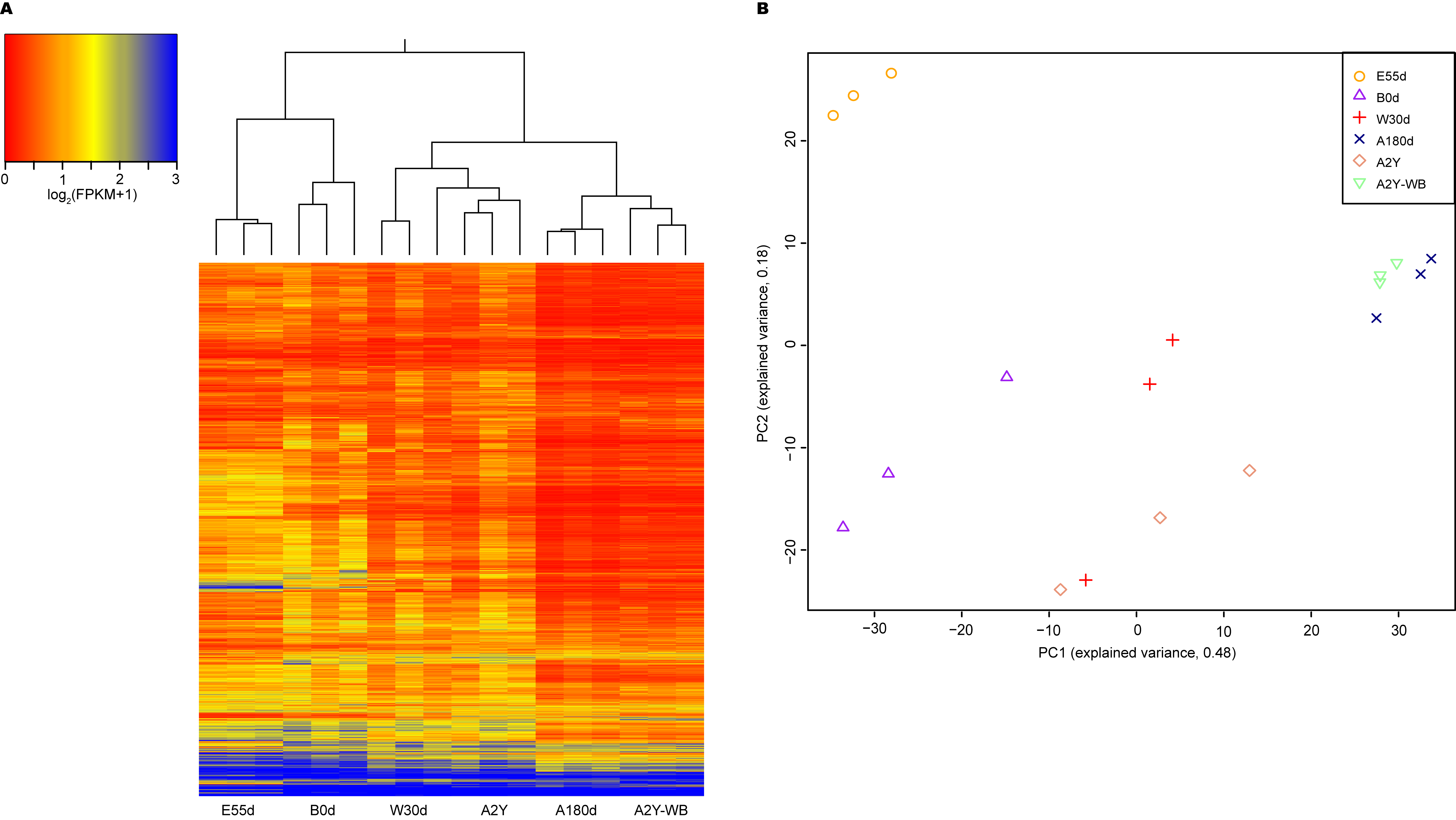

Supplement: S4 Fig — (A) Heat map shows the expression profile of lncRNAs. The top panel shows the tree constructed by Pearson correlation. (B) Two-way PCA plot of lncRNAs based on expression profile. (TIF) [file pone.0193552.s004.tif]
