# Supplementary material for: Long non-coding RNAs and mRNAs profiling during spleen development in pig
Source: PLoS One. 2018 Mar 14;13(3):e0193552. doi: 10.1371/journal.pone.0193552 (PMC5851557; doi:10.1371/journal.pone.0193552)
Supplement: S1 Table — (DOCX) [file pone.0193552.s005.docx]

**S1 Table. Summary of samples for total RNA sequencing**

| **Sample** | **Raw data (Gb)** | **High quality data (Gb)** | **Overall alignment rate (%)** |
| --- | --- | --- | --- |
| E55d-1 | 12.37 | 12.03 | 72.1 |
| E55d-2 | 12.95 | 12.41 | 72.3 |
| E55d-3 | 10.61 | 10.21 | 74.1 |
| B0d-1 | 12.46 | 12.20 | 72.2 |
| B0d-2 | 12.34 | 12.14 | 72.9 |
| B0d-3 | 12.93 | 12.73 | 71.6 |
| W30d-1 | 13.74 | 13.57 | 73.3 |
| W30d-2 | 10.45 | 10.32 | 73.3 |
| W30d-3 | 12.51 | 12.29 | 73.5 |
| A180d-1 | 11.48 | 11.19 | 77.3 |
| A180d-2 | 11.10 | 10.82 | 75.7 |
| A180d-3 | 10.48 | 10.07 | 75.7 |
| A2Y-1 | 16.84 | 16.72 | 76.1 |
| A2Y-2 | 20.85 | 20.77 | 77.6 |
| A2Y-3 | 16.04 | 14.12 | 73.9 |
| A2Y-WB-1 | 10.86 | 10.44 | 76.5 |
| A2Y-WB-2 | 10.50 | 10.10 | 76.3 |
| A2Y-WB-3 | 10.40 | 10.06 | 74.7 |
| Total | 228.91 | 222.19 | - |
